# Protective potential of the gallbladder in primary sclerosing cholangitis

**DOI:** 10.1016/j.jhepr.2022.100649

**Published:** 2022-12-17

**Authors:** Nora Cazzagon, Ester Gonzalez-Sanchez, Haquima El-Mourabit, Dominique Wendum, Dominique Rainteau, Lydie Humbert, Christophe Corpechot, Olivier Chazouillères, Lionel Arrivé, Chantal Housset, Sara Lemoinne

**Affiliations:** 1 Sorbonne Université, INSERM, Centre de Recherche Saint-Antoine (CRSA), Paris, France; 2 Department of Surgery, Oncology and Gastroenterology, University of Padova, Padova, Italy; 3 European Reference Network on Hepatological Diseases (ERN RARE-LIVER), Azienda Ospedale-Università Padova, Padova, Italy; 4 TGF-β and Cancer Group. Oncobell Program, Bellvitge Biomedical Research Institute (IDIBELL), L’Hospitalet De Llobregat, Barcelona, Spain; 5 Oncology Program, Ciberehd, National Biomedical Research Institute on Liver and Gastrointestinal Diseases, Instituto De Salud Carlos III, Spain; 6 Department of Physiological Sciences, Faculty of Medicine and Health Sciences, University of Barcelona, Spain; 7 Assistance Publique-Hôpitaux de Paris (AP-HP). Sorbonne Université. Department of Pathology, Saint-Antoine Hospital, Paris, France; 8 Assistance Publique-Hôpitaux de Paris (AP-HP). Sorbonne Université. Department of Clinical Metabolomics, Saint Antoine Hospital, Paris, France; 9 Assistance Publique-Hôpitaux de Paris (AP-HP). Sorbonne Université. Department of Hepatology, Reference Center for Inflammatory Biliary Diseases and Autoimmune Hepatitis (CRMR MIVB-H), ERN RARE-LIVER, Saint-Antoine Hospital, Paris, France; 10 Assistance Publique-Hôpitaux de Paris (AP-HP). Sorbonne Université. Department of Radiology, Saint-Antoine Hospital, Paris, France

**Keywords:** *Abcb4* knockout mice, Bile acids, Cholecystectomy, Gallbladder volume, Magnetic resonance imaging, ABC, ATP-binding cassette transporter, BA, bile acid, C4, 7α-hydroxy-4-cholesten-3-one, CFTR, cystic fibrosis transmembrane conductance regulator, CK19, cytokeratin 19, FGF19, fibroblast growth factor 19, IBD, inflammatory bowel disease, HPLC-MS/MS, high-performance liquid chromatography coupled to tandem mass spectrometry, MRC, magnetic resonance cholangiography, PSC, primary sclerosing cholangitis, UDCA, ursodeoxycholic acid, ULN, upper limit of normal

## Abstract

**Background & Aims:**

Gallbladder enlargement is common in patients with primary sclerosing cholangitis (PSC). The gallbladder may confer hepatoprotection against bile acid overload, through the sequestration and cholecystohepatic shunt of bile acids. The aim of this study was to assess the potential impact of the gallbladder on disease features and bile acid homeostasis in PSC.

**Methods:**

Patients with PSC from a single tertiary center who underwent liver MRI with three-dimensional cholangiography and concomitant analyses of serum bile acids were included. Gallbladder volume was measured by MRI and a cut-off of 50 ml was used to define gallbladder enlargement. Bile acid profiles and PSC severity, as assessed by blood tests and MRI features, were compared among patients according to gallbladder size (enlarged *vs*. normal-sized) or presence (removed *vs.* conserved). The impact of cholecystectomy was also assessed in the *Abcb4* knockout mouse model of PSC.

**Results:**

Sixty-one patients with PSC, all treated with ursodeoxycholic acid (UDCA), were included. The gallbladder was enlarged in 30 patients, whereas 11 patients had been previously cholecystectomized. Patients with enlarged gallbladders had significantly lower alkaline phosphatase, a lower tauro-*vs.* glycoconjugate ratio and a higher UDCA *vs*. total bile acid ratio compared to those with normal-sized gallbladders. In addition, gallbladder volume negatively correlated with the hydrophobicity index of bile acids. Cholecystectomized patients displayed significantly higher aspartate aminotransferase and more severe bile duct strictures and dilatations compared to those with conserved gallbladder. In the *Abcb4* knockout mice, cholecystectomy caused an increase in hepatic bile acid content and in circulating secondary bile acids, and an aggravation in cholangitis, inflammation and liver fibrosis.

**Conclusion:**

Altogether, our findings indicate that the gallbladder fulfills protective functions in PSC.

**Impact and implications:**

In patients with primary sclerosing cholangitis (PSC), gallbladder status impacts on bile acid homeostasis and disease features. We found evidence of lessened bile acid toxicity in patients with PSC and enlarged gallbladders and of increased disease severity in those who were previously cholecystectomized. In the *Abcb4* knockout mouse model of PSC, cholecystectomy causes an aggravation of cholangitis and liver fibrosis. Overall, our results suggest that the gallbladder plays a protective role in PSC.

## Highlights

• Gallbladder enlargement is associated with alleviated cholestasis in patients with PSC.
• Gallbladder volume is negatively correlated with bile acid hydrophobicity in PSC.
• Cholecystectomized patients with PSC display more severe cholangiographic features.
• Cholecystectomy aggravates cholangitis and liver fibrosis in *Abcb4* knockout mice.

## Graphical abstract

**Figure:**
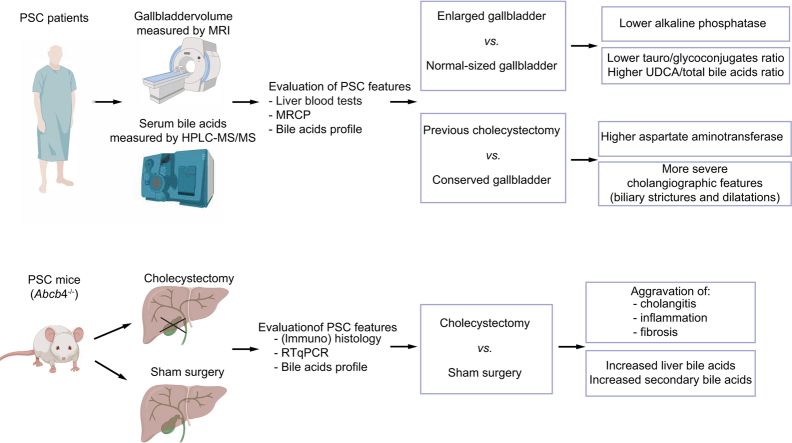
Impact and implications: In patients with primary sclerosing cholangitis (PSC), gallbladder status impacts on bile acid homeostasis and disease features. We found evidence of lessened bile acid toxicity in patients with PSC and enlarged gallbladders and of increased disease severity in those who were previously cholecystectomized. In the *Abcb4* knockout mouse model of PSC, cholecystectomy causes an aggravation of cholangitis and liver fibrosis. Overall, our results suggest that the gallbladder plays a protective role in PSC.

## Introduction

Primary sclerosing cholangitis (PSC) is a chronic cholestatic liver disease, characterized by fibro-inflammatory strictures and dilatations of the intra- and/or extrahepatic bile ducts, often associated with inflammatory bowel disease (IBD).^1^ The clinical course of the disease, although variable, is often progressive, leading to biliary cirrhosis and/or cancer.[2], [3], [4] Median transplant-free survival ranges from 13 years in patients with PSC referred to tertiary centers, to 21 years in population-based cohorts.^2^^,^^3^ The only medical treatment is ursodeoxycholic acid (UDCA), whose efficacy in PSC is limited and debated, meaning that liver transplantation remains the only life-extending therapeutic option in patients with end-stage liver disease. Several prognostic models including biochemical, histological and radiological features of the disease have been developed.^1^ Alkaline phosphatase, despite individual variation, is the most widely used serum marker of prognosis in PSC.[5], [6], [7], [8], [9] In our previous work, we demonstrated the prognostic value of MRI.^10^^,^^11^

The gallbladder, an accessory organ of the biliary tract, displays frequent abnormalities in PSC. First, the gallbladder is commonly enlarged in PSC.^12^^,^^13^ On the basis of ultrasound examination, it was previously shown that fasting gallbladder volume was increased by more than twofold in patients with PSC compared to healthy controls and patients with primary biliary cholangitis, cirrhosis of other aetiology or ulcerative colitis.^12^ A significant increase in gallbladder volume was also shown by MRI in patients with PSC compared to healthy controls.^13^ Other intrinsic abnormalities of the gallbladder in PSC have been reported at a prevalence of 41% and include gallstones, cholecystitis and mass lesions, *i.e.* polyps.^14^^,^^15^ As a consequence, it is relatively common for patients with PSC to undergo cholecystectomy.^4^ Most notably, due to the malignant nature of polyps in half of patients with PSC, cholecystectomy is now recommended for patients with gallbladder polyps ≥8 mm, preferably at an experienced center for patients with advanced disease.[15], [16], [17], [18]

The gallbladder is a well-conserved organ in vertebrates, which stores and concentrates bile between meals.^19^ Besides its role in physiology, there is evidence to indicate that the gallbladder exerts protective functions against bile acid overload, through the sequestration and/or a cholecystohepatic shunt of bile acids.^19^^,^^20^ We previously showed, in a model of cystic fibrosis transmembrane conductance regulator (*Cftr*)-deficient mice with enlarged gallbladders, that the enterohepatic recirculation of bile acids was reduced and consequently the formation of secondary toxic bile acids decreased.^20^ Conversely, cholecystectomy causes an increase in the enterohepatic recirculation of bile acids, and in the formation of secondary bile acids.[19], [20], [21] It was recently demonstrated that the gallbladder volume increased significantly following bile duct ligation in mice, whereas cholecystectomy caused more severe bile duct dilatation and cholestatic liver injury in this model.^22^ It was concluded from this study that the gallbladder alleviates hyperpressure caused by obstruction in the biliary tract.^22^

The aim of the present study was to assess the potential impact of gallbladder enlargement or removal on disease features and bile acid homeostasis in patients with PSC. We also assessed the effects of cholecystectomy in ATP-binding cassette transporter B4 (*Abcb4*) knockout mice, a well-established model of PSC and chronic cholestatic liver injury.[23], [24], [25]

## Patients and methods

### Patient study design

Patients with PSC followed at the reference center for inflammatory biliary diseases and autoimmune hepatitis in Saint-Antoine Hospital, Paris, France, between January 2004 and December 2013, who underwent both MRI with a three-dimensional magnetic resonance cholangiography (3D-MRC) and blood sampling, to allow for mass spectrometry analysis of bile acids, less than 15 days apart, were included and their records retrospectively reviewed. The date of blood sampling was used to define the inclusion date. The research was conducted in accordance with the 2013 Declaration of Helsinki and the 2018 Declaration of Istanbul and was approved by the Saint-Antoine Hospital institutional review committee. Written informed consent was provided by all participants. The diagnosis of PSC was based on typical macroscopic imaging (*i.e.*, multiple strictures of the large intra- and/or extrahepatic bile ducts on 3D-MRC).^17^^,^^26^ Exclusion criteria were a diagnosis of small-duct PSC, secondary sclerosing cholangitis or immunoglobin G4-associated cholangitis and a history of liver transplantation before inclusion. Sixty-one patients, including 11 patients who were previously cholecystectomized, were thus included. Fasting gallbladder volume was calculated by the analysis of MRI as described below. It was previously reported that median fasting gallbladder volume measured by MRI was 67 ml in patients with PSC and 32 ml in healthy controls and the threshold of 50 ml represented a clear cut-off between patients with PSC and healthy controls (75% of patients with PSC displayed a gallbladder volume above 50 ml and 75% of healthy controls displayed a gallbladder volume below 50 ml).^13^ Therefore, gallbladders whose volume reached or exceeded 50 ml were considered unambiguously enlarged, and patients whose gallbladder volume was < or ≥50 ml, were separated into two groups. Patients’ data at the time of inclusion were compared between the two groups. The Mayo and Amsterdam-Oxford risk scores were calculated as previously described.^27^^,^^28^ Percutaneous liver biopsy was performed in 38 patients, mostly before the inclusion MRI, and liver fibrosis was staged on a 0-to-4 scale, according to a METAVIR-derived scoring system.^29^^,^^30^ The diagnosis of cirrhosis required one of the following criteria: fibrosis stage F4 on liver biopsy, liver stiffness measured by vibration-controlled transient elastography ≥14.4 kPa^30^ or presence of liver dysmorphy associated with signs of portal hypertension (*i.e.*, platelet count <150 × 10^9^/L, presence of esophageal or gastric varices).

### MRI

MRI with 3D-MRC was performed as previously described by our group and in compliance with the International PSC study group (IPSCSG) recommendations.^31^^,^^32^ The procedure was preceded by fasting for at least 4 h. An axial T2-weighted single-shot fast spin-echo was performed. A 3D fat-suppressed T1-weighted gradient-echo sequence was performed before and after the administration of 20 ml of Gd-DOTA (Dotarem, Guerbet, Aulnay-sous-Bois, France), for hepatic arterial, portal venous, and equilibrium phase acquisition (at 30 s, 80 s and 3 min, respectively). MRC was performed with a free-breathing 3D high-spatial-resolution fast spin echo sequence. Native images and 3D maximum intensity projection reconstructions were analyzed on a workstation, using the Carestream Picture Archiving and Communication System (version 11.32; Carestream Health, Rochester, NY). Maximum intensity projections were analyzed on thick slabs of 10 or 20 mm, orientated in the acquisition plane. In patients with conserved gallbladders, the fasting gallbladder volume was calculated as previously described^13^ with modifications, integrating the area of gallbladder cross-sections obtained at 5 mm intervals on T2-weighted images (Fig. S1). Moreover, in order to assess the stability of gallbladder volume over time in the same patient, we repeated the measurements of gallbladder volume in at least three subsequent annual MRI sessions. The presence of gallstones, gallbladder polyps or cholecystitis was also assessed. Other MRC features including bile duct strictures or dilatations, cystic duct stenosis, parenchymal enhancement heterogeneity or dysmorphy, and portal hypertension were scored in all patients as previously described.^31^ The two ANALI scores, with and without gadolinium administration, which were associated with radiologic progression and clinical outcome in our previous studies,^10^^,^^31^ were also calculated.

### Mouse study design

*Abcb4*^*-/-*^ mice and *Abcb4*^*+/+*^ littermates were bred, using *Abcb4*^*+/-*^ heterozygous mice on an FVB/N genetic background (FVB·129P2-Abcb4tm1Bor/J) provided by Sanofi R&D (Chilly-Mazarin, France). Mice were housed at the CRSA animal facility (Institutional Animal Care and Use Direction, DDPP agreement No. C 75-12-01), in a temperature-controlled, specific pathogen-free environment, on a 12-hour light-dark cycle, with free access to standard chow (LASQCdiet® Rod16-R, Genobios, Laval, France) and water. All animals received humane care according to the criteria outlined in the “Guide for the Care and Use of Laboratory Animals” and experiments were approved by the Ethics Committee in Animal Experiments Charles Darwin No.5 (No. APAFIS#1600-201509021149154 v3).

Gallbladder volume was assessed in 4-week-old male *Abcb4*^*+/+*^ and *Abcb4*^*−/−*^ mice, who underwent cholecystectomy under isoflurane anesthesia, following 4-hour fasting. After cholecystectomy, gallbladders were weighted and their volume determined gravimetrically, assuming a density of 1 g/ml. The impact of gallbladder removal on the phenotypic traits of PSC was assessed in 7-week-old male *Abcb4*^*−/−*^ mice who underwent cholecystectomy or sham operations as previously described.^20^ Features of cholangiopathy are well established in *Abcb4*^-/-^ mice at the age of 7 weeks^25^**.** Five weeks after surgery, mice were sacrificed under isoflurane anesthesia, blood was drawn from the vena cava and the liver and spleen were collected and weighed. Liver samples were harvested following recommendations from the IPSCSG.^33^

### Bile acid analyses

Bile acids were analyzed using high-performance liquid chromatography coupled to tandem mass spectrometry (HPLC-MS/MS), in the serum from fasted patients and in the liver and plasma from unfasted mice, as previously described,^20^^,^^34^ using a Q-Trap 5500. All chemicals and solvents were of the highest purity available. Cholic acid, deoxycholic acid, chenodeoxycholic acid, UDCA, lithocholic acid, hyocholic acid, hyodeoxycholic acid and their glyco- and tauro-derivatives were obtained from Sigma-Aldrich (Saint Quentin Fallavier, France); 3-sulfate derivatives were a generous gift from J Goto (Niigita University of Pharmacy and Applied Life Science, Niigata, Japan); 23-Nor-5β-cholanoic acid-3α,12β diol, all muricholic acids, and their glyco and tauro derivatives were purchased from Steraloids Inc (Newport, USA). Acetic acid, ammonium carbonate, ammonium acetate and methanol, all of HPLC grade, were purchased from Sigma-Aldrich. The hydrophobicity index was determined based on the concentration and retention time of the different bile acids along a methanol gradient on a C18 column, lithocholic acid and tauroursodeoxycholic acid-3 S, displaying the highest and lowest retention time, respectively.

### FGF19 and C4 assays

Concentrations of fibroblast growth factor-19 (FGF19) were measured in serum samples collected from patients with PSC for bile acid analysis, using a sandwich ELISA (FGF19 Quantikine® ELISA kit, R&D Systems, Minneapolis, MN, USA). 7α-hydroxy-4-cholesten-3-one (C4) was measured using HPLC-MS/MS.

### Mouse liver (immuno)histology

Formalin (4%)-fixed, paraffin-embedded mouse liver tissue samples were cut into 4 μm-thick sections. Ductular reaction, liver inflammation and fibrosis, the hallmarks of cholangiopathy in the *Abcb4*^*-/-*^ mice, were assessed using cytokeratin 19 (CK19), F4/80 immunostaining and Sirius red staining, respectively. Liver tissue sections were stained with H&E or Sirius red or immunostained for CK19 or F4/80, using an anti-CK19 antibody (TROMA III, Developmental Studies Hybridoma Bank, Iowa University, IA, USA) and an anti-F4/80 antibody (SP115, Abcam, Cambridge, UK), respectively.

Stained sections were scanned on a virtual slide scanner (Hamamatsu, Tokyo, Japan) 2.0 HT, using a 3-charge-coupled device, time-delay integration camera with a resolution of 1.84 μm/pixel (x20 objective) and 0.92 μm/pixel (x40 objective). Morphometric analyses were performed blinded, using Image J analysis software (National Institutes of Health, Bethesda, MD, USA). H&E-stained liver tissue sections were blindly analyzed by a senior pathologist expert in liver histopathology (D.W.) following the Nakanuma scoring system, which was previously shown to have strong predictive value in PSC.^35^ The following histopathologic features were assessed in 10 portal spaces semi-quantitatively on a 0-3 scale: cholangitis and hepatitis activities, fibrosis stage and bile duct loss. Due to the specific characteristics of liver damage in *Abcb4*^*-/-*^ mice, grading criteria for cholangitis activity were adapted as previously described.^25^ Briefly: grade 0: no activity; grade 1: one bile duct with polymorphonuclear cell infiltrate; grade 2: two or more bile ducts with polymorphonuclear cell infiltrate; grade 3: presence of fibro-obliterative cholangitis.

### Quantitative reverse-transcription PCR

Total RNA was extracted from mouse liver tissue using RNeasy columns (Qiagen, Courtaboeuf, France). Complementary DNA was synthesized from total RNA (1 μg), using the SuperScript II Reverse Transcriptase (Thermo Fisher Scientific), and qPCR was performed on a Light-Cycler 96 (Roche Diagnostics, Basel, Switzerland), using Sybr Green Master Mix (Roche Diagnostics). The mRNA levels of target genes were normalized for those of *Hprt1* (hypoxanthine phosphoribosyltransferase 1) and expressed as relative levels (2^-ΔΔCt^). Primer sequences are provided in Table S1. The following genes were evaluated: *Tnfα* (tumor necrosis factor-α), a proinflammatory cytokine typically produced by reactive cholangiocytes in PSC,^36^ *Acta2* (α-smooth muscle actin) and *Col1a1* (collagen 1a1), which are fibrosis markers, and the genes encoding the main proteins involved in bile acid homeostasis, including *Cyp7a1* (cytochrome P450 7A1) and *Slc10a1* (sodium taurocholate cotransporting polypeptide), which are downregulated in cholestasis.^37^^,^^38^

### Statistical analyses

Patients’ characteristics are reported either as median (IQR), or as absolute number (percentage). Graphically, they are presented as box-whisker plots. To overcome potential assay variability, biochemical variables were expressed as ratios of upper limits of normal (ULN). Comparisons between two groups of patients defined according to gallbladder size (≥*vs.* <50 ml) or gallbladder presence (*vs.* cholecystectomy) were performed using chi-square test or Fisher’s exact test for categorical variables and Mann-Whitney test or Student’s *t* test for continuous variables, as appropriate. Potential correlations between gallbladder volume and any other continuous parameter were assessed using the Spearman coefficient. Quantitative data on mice were presented as box-whisker plots, and compared between cholecystectomized and sham-operated mice, using Student’s *t* test. In mouse studies, survival rates were calculated based on the Kaplan-Meier estimates and Log-rank test was used to assess if survival differed between groups. Statistical analyses of patients’ data were performed using IBM SPSS, Statistics v. 24 and subsequent versions. Mice data were analyzed using GraphPad Prism software version 5.0 (GraphPad Software San Diego, CA, USA). *p* values <0.05 were considered significant.

## Results

### Characteristics of the PSC study population

The study population comprised 61 patients (42 males and 19 females) with PSC. Eleven of them had been cholecystectomized before the date of inclusion for complications of gallstone disease and a suspicion of gallbladder cancer in five and two cases, respectively; the justification for cholecystectomy was unknown in the remaining four patients. The MRI at inclusion was used to measure gallbladder volume in the patients whose gallbladders were conserved at that time. It was performed for diagnostic purposes in five (8.2%) patients, and during follow-up in the remaining 56 (91.8%). All patients were taking UDCA (10 to 25 mg/kg/day) at the time of inclusion. Patients were aged 33[20], [21], [22], [23], [24], [25], [26], [27], [28], [29], [30], [31], [32], [33], [34], [35], [36], [37], [38], [39], [40], [41], [42] years at the time of PSC diagnosis, and 36 (25–52) years at the time of inclusion. IBD was present in 50 (82.0%) patients, including 31 with ulcerative colitis, 16 with Crohn’s disease and three with undetermined IBD. No patient had active ileal inflammation at the time of inclusion. Additional disease features are shown in Table 1.

**Table 1:** **Disease features in the PSC study population**.

	Patients with PSC, N = 61
Features at diagnosis	
Age, years	33 (20-42)
Male sex	42 (69)
IBD	50 (82)
PSC localization	
Intrahepatic only	15 (24.6)
Intra + extrahepatic	45 (73.8)
Extrahepatic only	1 (1.6)
Liver biopsy	40 (65.6)
Fibrosis according METAVIR	
F0	7 (17.5)
F1	12 (30)
F2	11 (27.5)
F3	8 (20)
F4	2 (5)
Features at inclusion	
Age, years	36 (25-52)
Time between diagnosis and inclusion, years	5.8 (2.2-10.0)
Total bilirubin, μmol/L	15.4 (11.0-36.3)
AST, xULN	1.4 (0.8-2.5)
GGT, xULN	2.9 (1.4-5.7)
ALP, xULN	1.5 (1.0-2.8)
PT, %	91 (78-100)
Serum albumin, g/L	40.3 (36.5-42.6)
Platelets, × 10^9^/L	283 (190-348)
Liver stiffness, kPa	10.8 (7.4-17.4)
Mayo risk score	-0.24 (-0.72 to 0.44)
Amsterdam-Oxford score at inclusion	1.72 (1.16-2.11)

Quantitative variables are expressed as median (IQR). Nominal variables are expressed as absolute number (%).

ALP, alkaline phosphatase; AST, aspartate aminotransferase; GGT, gamma-glutamyl transferase; PSC, primary sclerosing cholangitis; PT, prothrombin time; ULN, upper limit of normal.

### Evaluation of gallbladder volume in the PSC study population

**Fig. 1:**
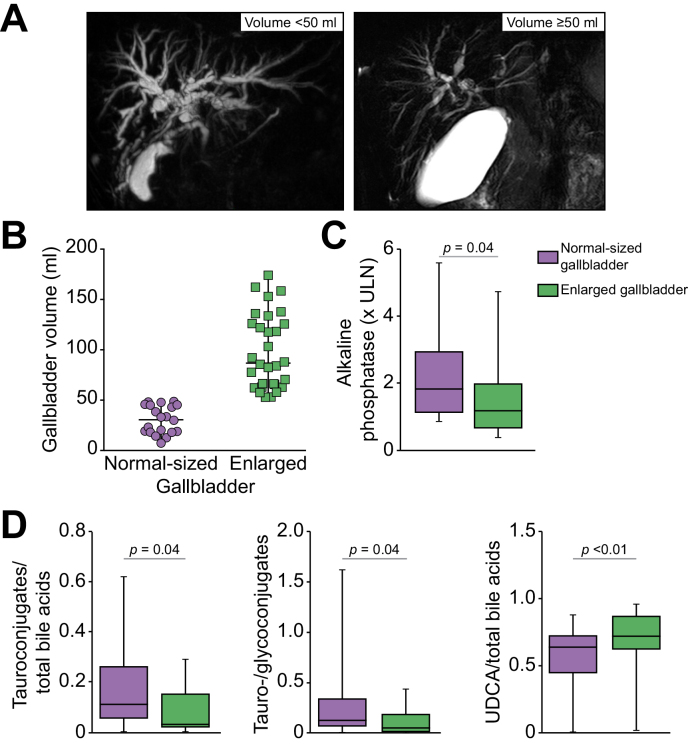
Gallbladder enlargement and related cholestatic profile in patients with PSC. (A) Gallbladder volume was measured on MRI in patients with PSC and conserved gallbladders, who were subsequently separated into two groups with enlarged (≥50 ml, n = 30) or normal-sized (<50 ml, n = 20) gallbladders, herein illustrated by gallbladder volumes of 11 and 103 ml, respectively. (B-D) Graphs showing the distribution of gallbladder volume (B), serum alkaline phosphatase levels (C), tauroconjugates/total BA ratios, Tauro-/glycoconjugate ratios and UDCA/total BA ratios (D), in the two groups. Levels of significance are indicated in the figure (Mann-Whitney test). A *p* value of less than 0.05 was considered significant.

In the study population, the gallbladder was present in 50 patients with PSC at the time of inclusion. Fasting gallbladder volumes measured by MRI analysis ranged between 7 ml and 174 ml in these patients. The median gallbladder volume was 62 (37–106) ml, which is consistent with the values of 67 ml and 55 ml reported in previous studies of gallbladder volume in PSC.^12^^,^^13^ Using a cut-off of 50 ml, we separated the 50 patients into two groups, *i.e.*, 20 and 30 patients whose fasting gallbladder volume was < and ≥ 50 ml respectively, as illustrated in Fig. 1A. The median values of gallbladder volume in the two groups were 31 (18–45) ml and 87 (65–128) ml, respectively (Fig. 1B). It should be noted that no cholecystitis, stricture of the cystic duct or obstructive gallbladder mass was detected among the patients with enlarged gallbladders (Table S2).

In addition, to evaluate the variability of gallbladder volume over time in an individual patient, we examined gallbladder volume on subsequent annual MRI in a subgroup of 36 (72%) patients, including 19 with enlarged gallbladders. This analysis showed that, within a range of 3 to 7 years, gallbladder volume was constantly less than or greater than 50 ml in an individual patient, with no overlap between the two groups (Fig. S2).

### Disease features in patients with PSC and enlarged gallbladders compared to those with normal-sized gallbladders

Whereas the clinical characteristics of patients with PSC with enlarged and normal-sized gallbladders were mostly similar, the serum levels of alkaline phosphatase were significantly lower in patients with enlarged gallbladders than in those with normal-sized gallbladders (Fig. 1C and Table S3). Except for gallbladder volume, we found no significant difference in the MRI features of patients whose gallbladders were enlarged or not (Table S2). We looked for a potential influence of gallbladder volume on the metabolism of bile acids, as assessed by their serum profile, and found that among the 50 patients with PSC whose gallbladders were conserved at the time of inclusion, the gallbladder volume was negatively correlated with the hydrophobicity index of circulating bile acids (ρ = −0.399, *p* <0.01). Moreover, gallbladder volume was positively correlated with the ratio of UDCA (ρ = 0.43, *p* <0.01) and of sulfoconjugates (ρ = 0.31, *p* = 0.03) *vs.* total bile acids*.* Analyses of serum bile acid profiles in patients with enlarged gallbladders compared to those with normal-sized gallbladders showed a lower ratio of tauroconjugates *vs.* glycoconjugates and *vs.* total bile acids and a higher ratio of UDCA *vs*. total bile acids (Fig. 1D and Table 2). We also observed trends towards lower cholic acid concentration and ratio *vs.* chenodeoxycholic acid, and towards a lower hydrophobicity index in patients with enlarged gallbladders (Table 2). FGF19 and C4 serum levels were available in only half of the patients in both groups and showed no difference between the two (Table 2). Importantly, potential confounding factors that could have affected bile acid metabolism were evenly distributed in the two groups. Notably, comparisons between the two groups revealed no difference in the prevalence of patients with IBD (all in clinical and endoscopic remission) or previous intestinal resection for IBD, nor in UDCA dosage at the time of inclusion (Table S4). We found no significant difference in adverse outcome-free survival (defined by survival without liver transplantation or cirrhosis decompensation) between the two groups (data not shown).

**Table 2:** **Serum bile acids in patients with normal-sized *vs.* enlarged gallbladders**.

	Normal-sized gallbladder (n = 20)	Enlarged gallbladder (n = 30)	*p* values
Total bile acids, μmol/L	35.41 (13.54-198.00)	33.49 (12.10-149.5)	0.64
Primary BA, μmol/L	5.76 (3.14-73.69)	4.48 (1.12-50.53)	0.10
CA, μmol/L	3.09 (1.02-31.37)	2.11 (0.28-19.86)	0.09
CDCA, μmol/L	3.29 (1.83-35.52)	2.61 (0.86-28.57)	0.21
CA/CDCA	0.74 (0.49-1.60)	0.62 (0.28-1.07)	0.07
Secondary BA, μmol/L	0.55 (0.08-1.65)	0.54 (0.09-1.27)	0.81
DCA, μmol/L	0.43 (0.06-1.48)	0.25 (0.09-1.16)	0.69
LCA, μmol/L	0.04 (0.01-0.29)	0.09 (0.00-0.29)	0.89
UDCA, μmol/L	18.09 (4.59-68.00)	28.4 (10.20-90.39)	0.38
Primary/secondary BA	13.19 (2.79-156.10)	10.95 (2.83-33.20)	0.48
Glycoconjugates, μmol/L	26.82 (11.91-151.43)	24.63 (8.87-120.08)	0.45
Tauroconjugates, μmol/L	2.40 (0.90-34.83)	0.86 (0.25-20.93)	0.14
Tauro-/glycoconjugates	0.13 (0.06-0.38)	0.06 (0.02-0.19)	**0.04**
UDCA/total BA	0.65 (0.49-0.73)	0.80 (0.65-0.90)	**<0.01**
Glycoconjugates/total BA	0.78 (0.70-0.88)	0.78 (0.68-0.87)	0.94
Tauroconjugates/total BA	0.12 (0.05-0.27)	0.04 (0.02-0.16)	**0.04**
Hydrophobicity index	0.82 (0.74-0.90)	0.77 (0.69-0.83)	0.09
FGF-19	132.84 (79.73-1,120.44)	129.76 (70.88-182.90)	0.44
C4	0.70 (0.20-1.34)	1.45 (0.20-2.38)	0.26

Quantitative variables are expressed as median (IQR).

Levels of significance are indicated in the table (Mann-Whitney test). A *p* value of less than 0.05 was considered significant.

BA, bile acid; C4, 7α-hydroxy-4-cholesten-3-one; CA, cholic acid; CDCA, chenodeoxycholic acid; DCA, deoxycholic acid; FGF-19, fibroblast growth factor-19; LCA, lithocholic acid; UDCA, ursodeoxycholic acid.

### Disease features in cholecystectomized patients compared to those with conserved gallbladders

**Fig. 2:**
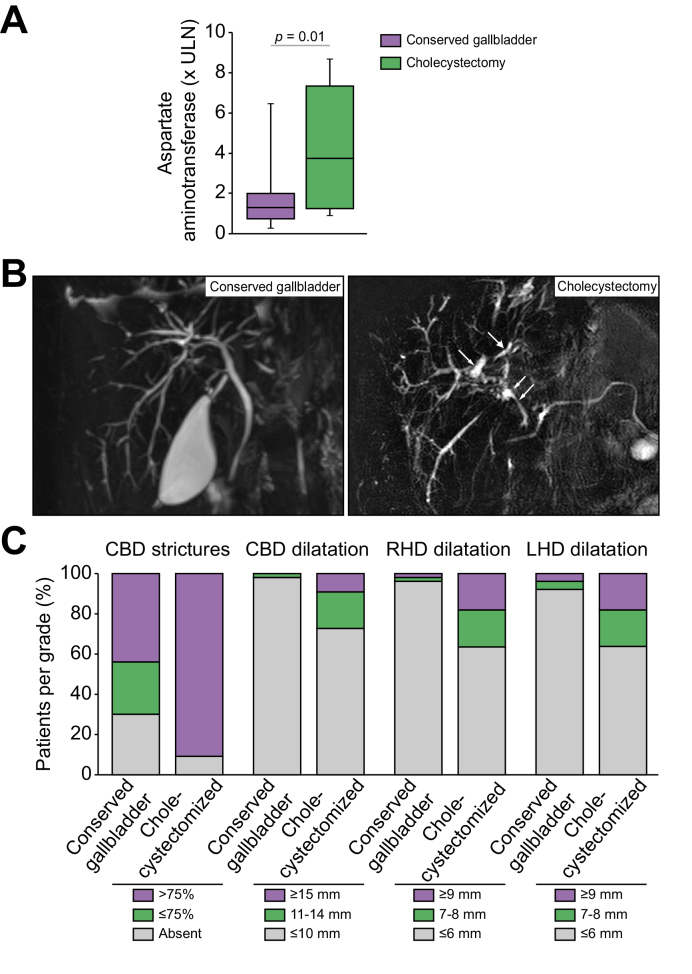
Impact of cholecystectomy on PSC features in patients. Comparison of (A) aspartate aminotransferase, (B–C) cholangiographic features at MRI including representative images (B) and quantitative analyses (C), in patients with PSC whose gallbladders were conserved (n = 50) or previously removed (n = 11). Levels of significance are indicated in the figure (Mann-Whitney test for A; Chi-squared test for C). A *p* value of less than 0.05 was considered significant. CBD, common bile duct; LHD, left hepatic duct; RHD, right hepatic duct.

Eleven patients in the PSC study population had been cholecystectomized prior to inclusion. Cholecystectomy was performed 3.4 (1.3–4.6) years before the date of inclusion on average. Compared to the non-cholecystectomized patients, patients who had been previously cholecystectomized showed similar clinical and biochemical characteristics, except for aspartate aminotransferase levels, which were significantly higher in these patients at inclusion (Fig. 2A and Table S5). In addition, severe MRI features were significantly more frequent at inclusion in these patients than in those with conserved gallbladders (Fig. 2B and Table 3). Thus, severe strictures (*p* = 0.02) and dilatations (*p <*0.01) of the common bile duct, severe dilatations of the right (*p* = 0.01) and left (*p* = 0.04) hepatic ducts and marked intrahepatic bile duct dilatations (*p* = 0.05) were all more frequent in cholecystectomized patients (Fig. 2C and Table 3). We also observed a trend towards a higher frequency of marked intrahepatic bile duct involvement (*p* = 0.09) and left hepatic duct enhancement (*p* = 0.09) in these patients (Table 3). Yet, no significant difference in serum bile acids was observed between the two groups, except for a trend towards higher levels of total bile acids and towards a lower ratio of UDCA *vs.* total bile acids in the group of cholecystectomized patients (Table S6). No difference in the adverse outcome-free survival was found between the two groups (data not shown).

**Table 3:** **MRI features in non-cholecystectomized *vs.* cholecystectomized patients**.

	Non-cholecystectomized (n = 50)	Cholecystectomized (n = 11)	*p* value
CBD strictures			**0.02**
Absent	15 (30.0)	1 (9.1)	
≤75%	13 (26.0)	0 (0)	
>75%	22 (44.0)	10 (90.9)	
CBD stricture length			0.21
≤2 mm	0 (0)	0 (0)	
3-10 mm	3 (6.0)	0 (0)	
>10 mm	32 (64)	10 (90.9)	
CBD dilatation			**<0.01**
≤10 mm	49 (98.0)	8 (72.7)	
11-14 mm	1 (2.0)	2 (18.2)	
≥15 mm	0 (0)	1 (9.1)	
CBD enhancement			0.60
Absent	34 (79.0)	6 (60.0)	
Thickness <2 mm	5 (11.6)	2 (20.0)	
Thickness 2-6 mm	4 (9.3)	2 (20.0)	
Thickness >6 mm	0 (0)	0 (0)	
RHD strictures			0.29
Absent	10 (20.0)	1 (9.1)	
≤75%	8 (16.0)	0 (0)	
>75%	31 (62.0)	10 (90.9)	
RHD stricture length			0.65
Absent	11 (2.0)	1 (9.1)	
≤2 mm	3 (6.0)	0 (0)	
3-10 mm	9 (18.0)	2 (18.2)	
>10 mm	26 (52.0)	8 (72.7)	
RHD dilatation			**0.01**
≤6 mm	47 (94.0)	7 (63.6)	
7-8 mm	1 (2.0)	2 (18.2)	
≥9 mm	1 (2.0)	2 (18.2)	
RHD enhancement			0.58
Absent	34 (79.1)	6 (60)	
Thickness <2 mm	5 (11.6)	1 (10)	
Thickness 2-6 mm	3 (7.0)	2 (20)	
Thickness >6 mm	0 (0)	1 (10)	
LHD strictures			0.33
Absent	13 (26.0)	1 (9.1)	
≤75%	8 (16.0)	1 (9.1)	
>75%	29 (58.0)	9 (81.8)	
LHD stricture length			0.45
Absent	14 (28.0)	1 (9.1)	
≤2 mm	4 (8.0)	1 (9.1)	
3-10 mm	8 (16.0)	1 (9.1)	
>10 mm	25 (48.0)	8 (72.7)	
LHD dilatation			**0.04**
≤6 mm	46 (92.0)	7 (63.6)	
7-8 mm	2 (4.0)	2 (18.2)	
≥9 mm	2 (4.0)	2 (18.2)	
LHD enhancement			0.09
Absent	35 (81.4)	5 (50)	
Thickness <2 mm	6 (14.0)	1 (10)	
Thickness 2-6 mm	2 (4.6)	3 (30)	
Thickness >6 mm	0 (0)	1 (10)	
IHBD Stricture			1.00
Absent	0 (0)	0 (0)	
≤75%	3 (6.0)	0 (0)	
>75%	47 (94.0)	11 (100)	
IHBD involvement			0.09
Absent	0 (0)	1 (9.1)	
≤25%	1 (2.0)	0 (0)	
>25%	49 (98.0)	10 (90.9)	
IHBD dilatation			0.05
None (≤3 mm)	15 (30.0)	1 (9.1)	
Mild (4 mm)	14 (28.0)	1 (9.1)	
Marked (≥5 mm)	21 (42.0)	9 (81.8)	
IHBD enhancement			0.27
Absent	31 (72.0)	5 (50)	
Thickness <2 mm	6 (13.9)	2 (20)	
Thickness 2-6 mm	6 (13.9)	1 (10)	
Thickness >6 mm	0 (0)	1 (10)	
Intraductal stones			1.00
Absent	34 (68.0)	7 (63.6)	
Present	16 (32.0)	4 (36.3)	
Dysmorphy			0.32
Absent	23 (46.0)	3 (27.3)	
Present	27 (54.0)	8 (72.7)	
Splenomegaly (splenic index > 480 cm^3^)			0.73
Absent	32 (61.6)	8 (72.7)	
Present	20 (38.4)	3 (27.3)	
Portal hypertension			0.73
Absent	34 (64.0)	8 (72.7)	
Present	18 (36.0)	3 (27.3)	
Parenchymal enhancement heterogeneity after GBCA			0.32
Absent	14 (32.6)	1 (10)	
Present	29 (67.4)	9 (90)	
ANALI score without gadolinium			0.61
0	14 (28.0)	1 (9.1)	
1	4 (8.0)	1 (9.1)	
2	5 (10.0)	1 (9.1)	
3	4 (8.0)	0 (0)	
4	13 (26.0)	5 (45.4)	
5	10 (20.0)	3 (27.3)	
ANALI score with gadolinium			0.32
0	13 (30.2)	1 (10)	
1	5 (11.6)	2 (20)	
2	25 (58.0)	7 (70)	

Nominal variables are expressed as absolute number (%).

Levels of significance are indicated in the table (Chi-squared test or Fisher’s exact test according to the frequencies, as appropriate). A *p* value of less than 0.05 was considered significant.

CBD, common bile duct; RHD, right hepatic duct; LHD, left hepatic duct; IHBD, intrahepatic bile duct; GBCA, gadolinium-based contrast agent.

Overall, these findings suggest that to some extent, the gallbladder exerted protective functions in patients with PSC, and even more so when the gallbladder is enlarged, whereas cholecystectomy was associated with more severe disease features. To address this latter possibility experimentally, we examined the direct impact of cholecystectomy on the phenotypic traits of PSC in the *Abcb4* knockout mouse model.

### Impact of cholecystectomy on phenotypic traits of PSC in the *Abcb4* knockout mice

**Fig. 3:**
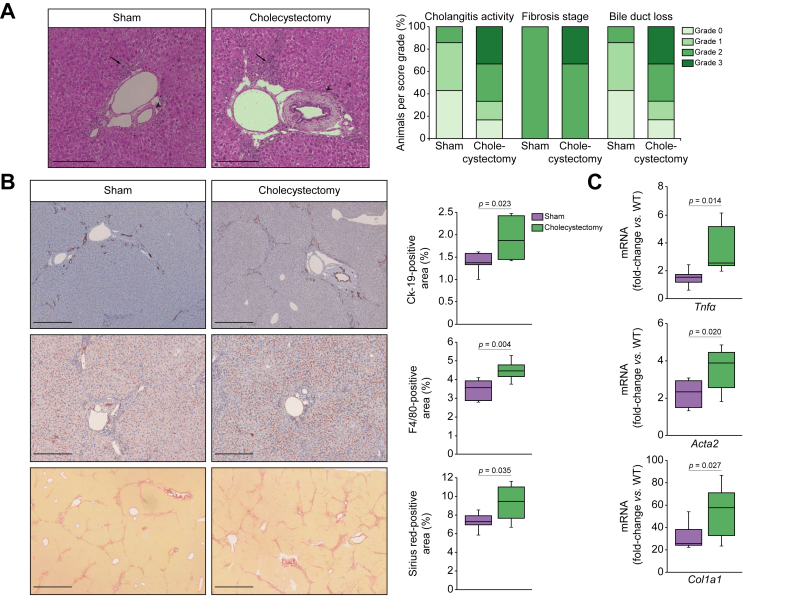
Impact of cholecystectomy on phenotypic traits of PSC in *Abcb4*^*-/-*^ mice. Liver samples were collected from Sham-operated (n = 7) or cholecystectomized (n = 6) *Abcb4*^*-/-*^ mice, 5 weeks after surgery, for (A) histological analysis of H&E-stained tissue sections (left panel, cholangitis characterized by periductal inflammation [arrows] and onion-skin fibrosis [arrowheads]), according to an adaptation of Nakanuma score (cholangitis activity, fibrosis score and bile duct loss were evaluated using a 0 to 3 scale (right panel, hepatitis activity=0 in all samples, not shown); (B) analysis of ductular reaction, inflammation and fibrosis, assessed by CK-19, F4/80 and Sirius red staining, respectively; (C) RT-qPCR analysis of proinflammatory (*Tnfα*) and profibrotic factor (*Col1a*1, *Acta2*) expression. Data were normalized for *Hprt1* and expressed relative to a pool of hepatic mRNA from WT mice. Levels of significance are indicated in the figure (Student’s *t* test). A *p* value of less than 0.05 was considered significant. Scale bars = 250 μm for H&E and F4/80; 500 μm for CK-19, 1 mm for Sirius red. RT-qPCR, quantitative reverse-transcription PCR; WT, wild-type.

**Fig. 4:**
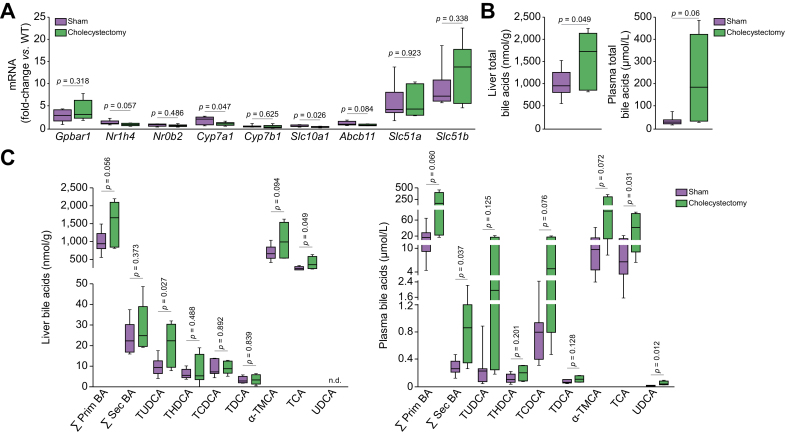
Impact of cholecystectomy on bile acid metabolism in *Abcb4*^*-/-*^ mice. Liver and plasma samples were collected from Sham-operated (n = 7) or cholecystectomized (n = 6) *Abcb4*^*-/-*^ mice, 5 weeks after surgery, for (A) RT-qPCR analysis of hepatic expression of genes involved in bile acid homeostasis (Data were normalized for *Hprt1* and expressed relative to a pool of hepatic mRNA from WT mice); (B–C) HPLC-MS/MS analysis of total bile acids (B) and individual bile acid species (C) in liver and plasma. Levels of significance are indicated in the figure (Student’s *t* test). A *p* value of less than 0.05 was considered significant. RT-qPCR, quantitative reverse-transcription PCR; WT, wild-type.

*Abcb4*^*-/-*^ mice showed no evidence of gallbladder enlargement (data not shown). Cholecystectomy was well tolerated and did not significantly affect animals’ survival or body weight gain compared to sham operations until the end of experiment (Fig. S3). Histological analysis by an expert pathologist showed that cholecystectomized mice developed more severe cholangitis, fibrosis and bile duct loss (Fig. 3A). Immunostaining analyses revealed that ductular reaction, liver inflammation and fibrosis were significantly more severe in cholecystectomized *Abcb4*^*-/-*^ mice than in sham-operated *Abcb4*^*-/-*^ mice (Fig. 3B). In keeping with these results, the hepatic expression of *Tnfα* was significantly higher in cholecystectomized *vs.* sham-operated *Abcb4*^*-/-*^ mice (Fig. 3C). Likewise, the hepatic expression of fibrosis markers, *i.e.*, *Acta2* and *Col1a*1, was significantly higher in cholecystectomized *Abcb4*^*-/-*^ mice (Fig. 3C). The hepatic expression of *Cyp7a1* and *Slc10a1* were both significantly decreased in cholecystectomized compared to sham-operated *Abcb4*^*-/-*^ mice (Fig. 4A). This implied that cholestasis was aggravated as a result of cholecystectomy in *Abcb4*^*-/-*^ mice, which was confirmed by a significant increase in total bile acid content in the liver of cholecystectomized *vs.* sham-operated *Abcb4*^*-/-*^ mice (Fig. 4B). The analyses of bile acid species showed that the concentrations of taurocholic acid were significantly increased both in the liver and plasma of cholecystectomized *Abcb4*^*-/-*^ mice compared to sham-operated mice (Fig. 4C). In addition, although both primary and secondary bile acids were increased in the plasma of cholecystectomized mice compared to sham-operated mice, only the elevation of secondary bile acids was statistically significant (Fig. 4C). Overall, these results clearly demonstrated that cholecystectomy caused an increase in bile acid overload and an aggravation of cholangiopathy in the *Abcb4* knockout mouse model of PSC.

## Discussion

In the present study, we provide several pieces of evidence indicating that the gallbladder promotes hepatobiliary protection in PSC. First, we show that, compared to patients with PSC and normal gallbladder volume, those with enlarged gallbladders have significantly lower alkaline phosphatase, which is associated with a favorable prognosis in PSC.^8^ We also show that, compared to patients with conserved gallbladders, those who underwent a cholecystectomy display more severe cholangiographic features and higher serum aspartate aminotransferase, which are both associated with adverse outcomes in PSC.^10^^,^^27^ Furthermore, we demonstrate that cholecystectomy causes an aggravation of cholangiopathy features in the *Abcb4* knockout mouse model of PSC, in support of a causal relationship between gallbladder removal and increased disease severity. Two main mechanisms may account for the protective functions of the gallbladder in PSC, *i.e.* a release of hyperpressure in the biliary tract,^22^ and a modulation of the enterohepatic circulation and composition of the bile acid pool,[19], [20], [21] which may ultimately restrict bile acid cytotoxicity. Both mechanisms can be amplified by gallbladder enlargement and suppressed by cholecystectomy.

To address the impact of gallbladder enlargement in PSC, we first measured fasting gallbladder volume using MRI in the largest number of patients with PSC to date. The threshold was set at 50 ml to define gallbladder enlargement on the basis of a previous MRI study, which included patients with PSC and healthy individuals. Based on these criteria, we found that the gallbladder was enlarged in more than half of patients with PSC. We did not detect a local cause of gallbladder distension, such as stenosis of the cystic duct, in any of these patients. We suspect that gallbladder enlargement is caused by hyperpressure in the biliary tract of patients with PSC, as recently shown to explain gallbladder enlargement in bile duct-ligated mice.^22^ However, the reason why some patients with PSC develop gallbladder enlargement whereas others do not is currently unknown. As in previous studies,^12^^,^^13^ we found no relationship between gallbladder enlargement on the one hand, and the severity of bile duct strictures, the stage of disease or the association with IBD, on the other hand. Additionally, we observed no relationship with the serum levels of FGF19, the hormone that stimulates gallbladder filling.^39^ However, this data was only available in half of our cohort. Gallbladder volume showed little or no variation over time in the same patient, which is of particular importance when considering the potential long-term effects of gallbladder enlargement. It was previously shown that both fasting and postprandial gallbladder volumes were enlarged in patients with PSC, whereas the ejection fraction in response to a non-physiological test meal was normal.^12^^,^^13^ In a similar situation, *i.e.*, *Cftr*-deficient mice, in which both fasting and postprandial gallbladder volumes were enlarged, we previously demonstrated that while the ejection fraction in response to a non-physiological stimulus (cholecystokinin-8) was normal, the enterohepatic circulation of bile acids and the formation of secondary bile acids were decreased.^20^ Likewise, in patients with PSC, we found that the gallbladder volume was negatively correlated with bile acid hydrophobicity. On the contrary, gallbladder volume was positively correlated with the ratio of UDCA to total bile acids. Therefore, decreased bile acid toxicity may have contributed to lessen cholestatic liver injury, as corroborated by lower alkaline phosphatase levels in patients with enlarged gallbladders compared to those with normal-sized gallbladders. In addition, patients with PSC with enlarged gallbladders displayed lower tauro-*vs.* glycoconjugate ratios compared to those with normal-sized gallbladders. Previous work demonstrated the prognostic value of circulating bile acid profiles in patients with PSC. Notably, lower tauro-*vs.* glycoconjugate ratios were shown to predict a lower risk of hepatic decompensation.^40^

We also evaluated the impact of cholecystectomy, as another approach to assess gallbladder-mediated protection in PSC. Patients with PSC whose gallbladders had been removed were characterized by an increased severity of cholangiographic features compared to patients whose gallbladders were conserved. Severe dilatations in particular were more frequent in the different portions of the biliary tract of cholecystectomized patients. This finding strongly suggests that cholecystectomy triggers an aggravation of hyperpressure in the biliary tract in PSC, as previously reported in bile duct-ligated mice.^22^ In keeping with this scenario, the serum levels of aspartate aminotransferase were significantly higher in cholecystectomized patients, as one would expect from an increase in the biliary infarcts that develop upstream of biliary obstructions. Serum bile acid analyses revealed no significant difference between cholecystectomized and non-cholecystectomized patients. However, we observed a trend towards higher levels of total bile acids in the group of cholecystectomized patients. In a small number of participants, we also observed trends towards lower C4 and higher FGF19 concentrations in this group, reflecting lower and higher rates of bile acid synthesis and ileal reabsorption, respectively. This is consistent with post-cholecystectomy acceleration of bile acid enterohepatic circulation.[19], [20], [21] We confirmed the deleterious effects of cholecystectomy on the phenotypic traits of PSC in the *Abcb4* knockout mice. All histopathological features of PSC, *i.e.* cholangitis, inflammation and liver fibrosis, were aggravated by cholecystectomy in this model. We showed that the deleterious effect of cholecystectomy involved an increase in the overload of bile acids, most notably taurocholate, which was previously shown to trigger biliary damage in different murine models, including *Abcb4* knockout mice.^41^^,^^42^ The enterohepatic circulation of bile acids was also likely accelerated, as indicated by an increased proportion of secondary bile acids in the circulation of *Abcb4* knockout mice post-cholecystectomy.

Thus, our present results infer that cholecystectomy may cause disease aggravation in patients with PSC. The deleterious effects of cholecystectomy on liver disease also likely apply to other pathological settings. A very large cohort study previously showed that independent of cholelithiasis, patients who underwent cholecystectomy were twice as likely to develop cirrhosis and to have elevated serum aminotransferases as those without previous cholecystectomy.^43^

We recognize that our study has some limitations. The main limitation relates to the restricted size of the PSC study population. Moreover, the significant differences we observed between patient groups were limited to surrogate markers, such as alkaline phosphatase or cholangiographic features. Larger studies are required to confirm our findings and to investigate the potential impact of gallbladder presence and size on the natural history of PSC.

The major strength of our study was the collection of data from patients with PSC and from *Abcb4*^-/-^ mice. Together, these data suggest that the gallbladder could provide hepatobiliary protection in PSC through the sequestration of bile and the modification of bile acid molecular species composition. These protective mechanisms are suppressed by cholecystectomy and to some extent amplified by gallbladder enlargement. More work remains to be done to fully elucidate the complex role of the gallbladder in PSC and in other chronic liver diseases.

## Financial support

This work was supported by funding from the Microbiome Foundation, PSC Partners Seeking a Cure, Agence Nationale de la Recherche (ANR) grant # 15-CE14-0007-01 and Fondation pour la Recherche Médicale (FRM) EQU202003010517. E.G-S. received post-doctoral fellowships from the Spanish Association for the Study of the Liver (AEEH), from the Alfonso Martin Escudero Foundation (FAME), and a post-doctoral contract from CIBEREHD, Instituto de Salud Carlos III.

## Authors’ contributions

NC, LA, CH and SL designed the study. NC, EGS, SL, HEM, DW, DR, LH, LA generated the data. NC, EGS, CH and SL analyzed the data. All authors critically revised and approved the final version of the manuscript.

## Data availability statement

The data that support the findings of this study are available on request from the corresponding author [SL, NC]. The data are not publicly available due to privacy restrictions.

## Conflict of interest

Nora Cazzagon, Ester Gonzalez-Sanchez, Haquima El-Mourabit, Dominique Wendum, Dominique Rainteau, Lydie Humbert, Olivier Chazouillères, Lionel Arrivé, Chantal Housset and Sara Lemoinne declare no conflict of interest related to this paper. Christophe Corpechot declares the following conflicts of interest: Intercept, Arrow, Cymabay, Ipsen, Calliditas, Gilead.

Please refer to the accompanying ICMJE disclosure forms for further details.
