# Supplementary material for: Protective potential of the gallbladder in primary sclerosing cholangitis
Source: JHEP Rep. 2022 Dec 17;5(4):100649. doi: 10.1016/j.jhepr.2022.100649 (PMC10009728; doi:10.1016/j.jhepr.2022.100649)
Supplement: Multimedia component 1 [file mmc1.pdf]

Protective potential of the gallbladder in primary sclerosing cholangitis

Nora Cazzagon, Ester Gonzalez-Sanchez, Haquima El-Mourabit, Dominique Wendum, Dominique Rainteau, Lydie Humbert, Christophe Corpechot, Olivier Chazouillères, Lionel Arrivé, Chantal Housset, Sara Lemoinne

Table of contents

Fig. S1..2

Fig. S2..2

Fig. S3..3

Table S1..4

Table S2..5

Table S3..8

Table S4..9

Table S5..10

Table S6..11

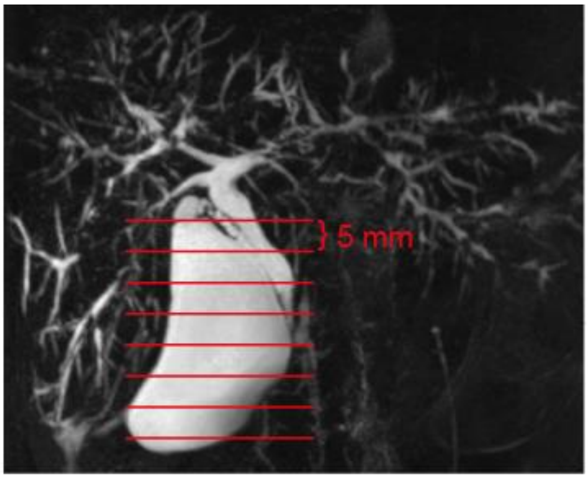

Fig. S1. Fasting gallbladder volume calculation. Fasting gallbladder volume was calculated in a 3D workstation using 3D segmentation and volume measurements integrating the area of gallbladder cross-sections obtained at 5-mm interval on T2-weighted images.

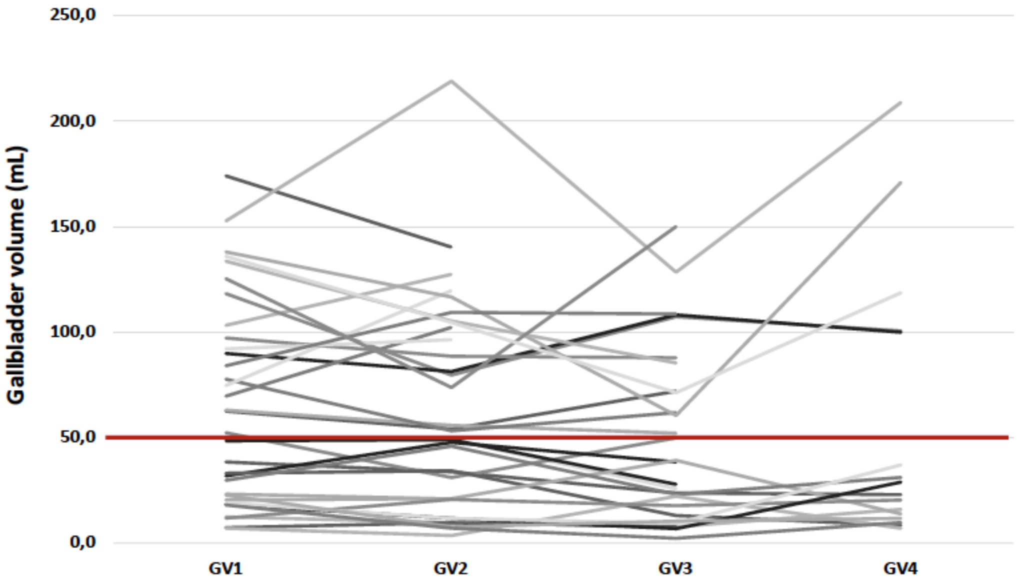

Fig. S2. Gallbladder volume variability over time. Gallbladder volume was measured in subsequent MRI from inclusion in a subgroup of PSC patients with conserved gallbladder. The red line represents the threshold of 50 mL chosen to subgroup patients with enlarged or non-enlarged. The graph showed that the gallbladder volume was constantly inferior or superior to 50 mL in an individual patient, with no overlap between the two groups.

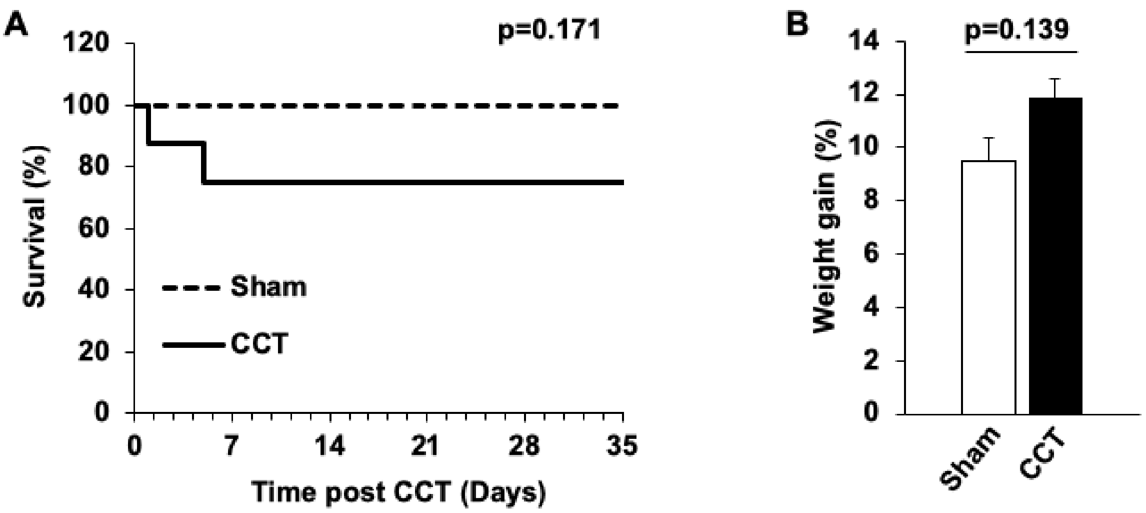

Fig. S3. Tolerance of cholecystectomy in Abcb4$^{-/-}$ mice. Cholecystectomy (CCT) was well tolerated as indicated by the analysis of survival, estimated by the Kaplan-Meier method (A), and the analysis of body weight gain (B) (n=-6-7 mice per group). Bar graphs represent means ± SEM. Levels of significance are indicated in the figure (Log-rank test for A; Student's *t* test for B). A *p* value of less than 0.05 was considered significant

Table S1. Primers used for qPCR

***Gene* (PROTEIN)**	**Forward**	**Reverse**	**GenBank Access No.**
Acta2 (α-SMA)	TCTATGCCTCTGGACGTACA	CCAGACGCATGATGGCATG	NM_007392
Abcb11 (BSEP)	ATCCTGCTTCTGGACATGGCTA	ATGGGCAACTGAGATGATTGTG	NM_021022
Ccl2	GCCTGCTGTTCACAGTTGC	CAGGTGAGTGGGGCGTTA	NM_011333
Col1a1	GAAACCCGAGGTATGCT GA	GACCAGGAGGACCAGGAAGT	NM_007742
Cyp7a1	ACTCTCTGAAGCCATGATGCAA	AGCGTTAGATATCCGGCTTCAA	NM_007824
Cyp7b1	TCCGAGAAGTGCAGGAGGAT	TTTCCGGGTCATTGTGTATGAG	NM_007825
Nr1h4 (FXR)	TCCGGACATTCAACCATCAC	TCACTGCACATCCCAGATCTC	NM_001163700
Hprt1	TCAGTCAACGGGGGACATAA	TGCTTAACCAGGGAAAGCAAA	NM_013556.2
Slc10a1 (NTCP)	ACCTCCTCCCTGATGCCTTT	GTTGGACGTTTTGGAATCCTG	NM_011387
Slc51a (OSTα)	GTCTCAAGTGATGAACTGCCA	TTGAGTGCTGAGTCCAGGTC	NM_145932
Slc51b (OSTβ)	GATGCGGCTCCTTGGAATTA	CGATTTCTGTTTGCCAGGATG	NM_178933
Nr0b2 (SHP)	CGATCCTCTTCAACCCAGATG	AGGGCTCCAAGACTTCACACA	NM_011850
Tgfb1	GTCAGACATTCGGGAAGCAG	GCGTATCAGTGGGGGTCA	NM_011577
Gpbar1 (TGR5)	GTC AGC TCC CTG TTC TTT GC	CAG GAG GCC ATA AAC TTC CA	NM_174985
Tnfα	ATGAGCACAGAAAGCATGATC	TACAGGCTTGTCACTCGAATT	NM_013693

Table S2. MRI features in patients with normal-sized *vs.* enlarged gallbladder

	Normal-sized gallbladder (n=20)		Enlarged gallbladder (n=30)		p
**Gallbladder characteristics**					0.47
• Gallstones (non-obstructive)	2	(10)	2	(6.7)	
• Polyps (non-obstructive)	0	(0)	2	(6.7)	
• Cholecystitis	0	(0)	0	(0)	
**Cystic duct**					0.40
• Normal	19	(95)	30	(100)	
• Abnormal	1	(5)	0	(0)	
**CBD strictures**					0.69
• Absent	7	(35)	8	(26.7)	
• ≤75%	4	(20)	9	(30)	
• >75%	9	(45)	13	(43.3)	
**CBD stricture length**					0.45
• ≤2 mm	0	(0)	0	(0)	
• 3-10 mm	2	(10)	1	(3.3)	
• >10 mm	11	(55)	21	(70)	
**CBD dilatation**					0.40
• ≤10 mm	19	(95)	30	(100)	
• 11-14 mm	1	(5)	0	(0)	
• ≥15 mm	0	(0)	0	(0)	
**CBD enhancement**					0.26
• Absent	14	(70)	20	(66.7)	
• Thickness <2 mm	2	(10)	3	(10)	
• Thickness 2-6 mm	3	(15)	1	(3.3)	
• Thickness >6 mm	0	(0)	0	(0)	
**RHD strictures**					0.64
• Absent	5	(25)	6	(16.7)	
• ≤75%	4	(20)	4	(13.3)	
• >75%	11	(55)	20	(66.7)	
**RHD stricture length**					0.35
• Absent	5	(25)	6	(20)	
• ≤2 mm	1	(5)	2	(6.7)	
• 3-10 mm	6	(30)	3	(10)	
• >10 mm	8	(40)	18	(60)	
**RHD dilatation**					0.41
• ≤6 mm	19	(95)	28	(93.3)	
• 7-8 mm	0	(0)	1	(3.3)	
• ≥9 mm	1	(5)	0	(0)	
**RHD enhancement**					0.30
• Absent	15	(75)	19	(63.3)	
• Thickness <2mm	2	(10)	3	(10)	
• Thickness 2-6 mm	2	(10)	1	(3.3)	
• Thickness >6 mm					
**LHD strictures**					0.63
• Absent	6	(30)	7	(23.3)	
• ≤75%	4	(20)	4	(13.3)	
• >75%	10	(50)	19	(63.3)	
**LHD stricture length**					0.80
• Absent	6	(30)	8	(26.7)	
• ≤2 mm	2	(10)	2	(6.7)	
• 3-10 mm	4	(20)	4	(13.3)	

• >10 mm	8	(40)	16	(53.3)	
**LHD dilatation**					0.48
• ≤6 mm	19	(95)	27	(90)	
• 7-8 mm	0	(0)	2	(6.7)	
• ≥9 mm	1	(5)	1	(3.3)	
**LHD enhancement**					0.16
• Absent	14	(70)	21	(70)	
• Thickness <2mm	3	(15)	3	(10)	
• Thickness 2-6 mm	2	(10)	0	(0)	
• Thickness >6 mm	0	(0)	0	(0)	
**IHBD Stricture**					1.00
• Absent	0	(0)	0	(0)	
• ≤75%	1	(5)	2	(6.7)	
• >75%	19	(95)	28	(93.3)	
**IHBD involvement**					1.00
• absent	0	(0)	0	(0)	
• ≤25%	0	(0)	1	(3.3)	
• >25%	20	(100)	29	(96.7)	
**IHBD dilatation**					0.45
• none (≤3 mm)	8	(40)	7	(23.3)	
• mild (4 mm)	5	(25)	9	(30)	
• marked (≥5 mm)	7	(35)	14	(46.7)	
**IHBD enhancement**					0.26
• Absent	12	(60)	19	(63.3)	
• thickness <2mm	3	(15)	3	(10)	
• thickness 2-6 mm	4	(20)	2	(6.7)	
• thickness>6 mm	0	(0)	0	(0)	
**Intraductal stones**					0.54
• Absent	15	(75)	19	(63.3)	
• Present	5	(25)	11	(36.7)	
**Dysmorphy**					0.39
• Absent	11	(55)	12	(40)	
• Present	9	(45)	18	(60)	
**Splenomegaly (splenic index > 480 cm^3)**					0.77
• Absent	13	(65)	17	(56.7)	
• Present	7	(35)	13	(43.3)	
**Portal Hypertension**					0.56
• Absent	14	(70)	18	(60)	
• Present	6	(30)	12	(40)	
**Parenchymal enhancement heterogeneity after GBCA**					0.05
Absent	9	(45)	5	(16.7)	
Present	10	(50)	19	(63.3)	
**ANALI score without gadolinium**					0.58
• 0	8	(40)	6	(20)	
• 1	1	(5)	3	(10)	
• 2	2	(10)	3	(10)	
• 3	2	(10)	2	(6.7)	
• 4	3	(15)	10	(33.3)	
• 5	4	(20)	6	(20)	
**ANALI score with gadolinium**					0.11
• 0	9	(45)	4	(13.3)	
• 1	1	(5)	4	(13.3)	
• 2	9	(45)	16	(53.3)	

Quantitative variables are expressed as medians (interquartile range). Nominal variables are expressed as absolute number (percentage). Abbreviations: CBD, common bile duct; RHD, right hepatic duct; LHD, left hepatic duct; IHBD, intrahepatic bile duct; GBCA, Gadolinium based contrast agent. Levels of significance are indicated in the table (Chi-squared test of Fisher test where appropriate). A *p* value of less than 0.05 was considered significant.

Table S3. Characteristics of patients with normal-sized *vs.* enlarged gallbladder

	**Normal-sized gallbladder** (n=20)	**Enlarged gallbladder** (n=30)	**p**
**Age at diagnosis**, years	25 (17-36)	33 (21-42)	0.21
**Male gender**, n(%)	12 (60)	23 (76.7)	0.23
**BMI**	21.6 (18.1-23.8)	23.0 (21.5-24.8)	0.32
**LD-PSC localization**			0.55
• Intrahepatic	5 (25)	10 (33.3)	
• Intra + extrahepatic	15 (75)	19 (63.3)	
• Extrahepatic	0 (0)	1 3.3)	
**IBD**	16 (80)	27 (90)	0.42
• Ulcerative colitis	10 (62.5)	15 (55.6)	0.91
• Crohn's disease	5 (31.3)	10 (37)	
• Indeterminate	1 (6.3)	2 (7.4)	
**Age**, years	30 (23-50)	36 (25-52)	0.61
**Interval diagnosis-inclusion**, years	7 (4-13)	5 (2-9)	0.24
**Cirrhosis**	7 (41.2)	9 (30.0)	0.75
**Liver stiffness**, kPa	11.9 (7.1-17.4)	10.1 (7.4-39.3)	0.71
**Total bilirubin**, µmol/L	21.4 (10.8-41.2)	13.5 (10.8-24.6)	0.28
**AST,** x ULN	1.8 (1.0-2.7)	1.2 (0.6-1.9)	0.11
**γGT**, x ULN	2.9 (2.0-14.7)	2.0 (0.9-5.5)	0.11
**ALP**, x ULN	1.8 (1.1-3.0)	1.2 (0.7-2.0)	**0.04**
**PT**, %	91 (86-99)	90 (74-100)	0.88
**Serum albumin**, g/L	40.6 (35.1-42.5)	40.0 (36.4-42.7)	0.95
**Platelets count** x 10^9/L	286 (234-346)	284 (173-347)	0.63
**Revised Mayo risk score**	0.01 (-0.72-1.42)	-0.39 (-0.90-0.22)	0.44
**Amsterdam Oxford risk score**	1.74 (1.34-2.45)	1.65 (1.15-2.05)	0.36

All parameters except age were determined at inclusion. Quantitative variables are expressed as median (interquartile range). Nominal variables are expressed as absolute number (percentage). Abbreviations: BMI, body mass index; LD-PSC, large-duct-PSC; IBD, inflammatory bowel disease; AST, aspartate aminotransferase; ULN, upper limit of normal; γGT, gamma-glutamyl transpeptidase; ALP, alkaline phosphatase; PT, prothrombin time. Revised Mayo score was calculated as described in Kim WR *et al.* Mayo Clin. Proc. 2000;75:688–694, Amsterdam-Oxford Model was calculated as described in de Vries EM et al. Gut. 2018;67:1864–1869. Levels of significance are indicated in the table (Mann-Whitney test for continuous variables; chi-squared test, or Fisher test where appropriate, for nominal variables). A *p* value of less than 0.05 was considered significant.

Table S4. Factors potentially affecting serum bile acids in patients with normal-sized *vs.* enlarged gallbladder

	**Normal-sized gallbladder** (n=20)	**Enlarged gallbladder** (n=30)	**p**
**IBD in remission at inclusion**	9 (56.3)	19 (63.3)	1.00
**Intestinal resection for IBD**	1 (6.3)	1 (3.3)	1.00
**UDCA dosage**			0.45
• 10-15 mg/Kg	7 (35)	7 (35)	
• 15-20 mg/Kg	9 (45)	9 (45)	
• 20-25 mg/Kg	1 (5)	4 (20)	

Nominal variables are expressed as absolute number (percentage). Levels of significance are indicated in the table (Chi-squared test of Fisher test where appropriate). A *p* value of less than 0.05 was considered significant.

Table S5. Characteristics of patients with conserved gallbladders *vs.* cholecystectomy

	Conserved gallbladder (n=50)	Cholecystectomy (n=11)	p
**Age at PSC diagnosis**, years	32 (20-41)	37 (25-43)	0.25
**Male gender**, n(%)	35 (70.0)	7 (63.6)	0.73
**BMI**	22.1 (19.8-24.8)	21.4 (20.4-24.2)	0.69
**PSC localization**			0.09
• Intrahepatic	15 (30)	0 (0)	
• Intra + extrahepatic	34 (68)	11 (100)	
• Extrahepatic	1 (2)	0 (0)	
**IBD**	43 (86)	7 (63.6)	0.10
• Ulcerative colitis	25 (58.1)	6 (85.7)	
• Crohn's disease	15 (34.9)	1 (14.3)	
• Indeterminate	3 (7)	0 (0)	
**Age at inclusion**, years	35 (24-51)	41 (25-59)	0.28
**Interval diagnosis-inclusion,** years	5 (2-10)	4 (1-13)	0.84
**Cirrhosis at inclusion**	16 (37.2)	3 (27)	1.00
**Liver stiffness**, kPa	11.3 (7.3-17.5)	10.6 (7.8-15.7)	0.81
**Total bilirubin**, µmol/L	14.5 (11.0-36.0)	26.5 (16.3-69.5)	0.15
**AST**, x ULN	1.3 (0.8-2.0)	3.8 (1.3-7.3)	**0.01**
**γGT**, x ULN	2.6 (1.4-5.5)	4.9 (3.7-11.8)	0.17
**ALP**, x ULN	1.3 (0.9-2.6)	2.1 (1.2-3.4)	0.26
**PT**, %	91 (80-100)	94 (70-106)	0.68
**Serum albumin**, g/L	40.1 (35.3-42.6)	40.3 (37.7-43.7)	0.74
**Platelets count**, x 10^9/L	286 (194-347)	276 (162-461)	0.70
**Revised Mayo risk score**	-0.36 (-0.80-0.38)	-0.11 (-0.61-)	0.82
**Amsterdam Oxford risk score**	1.71 (1.16-2.09)	1.8 (1.1-)	0.94

Quantitative variables are expressed as medians (interquartile range). Nominal variables are expressed as absolute number (percentage). Abbreviations: BMI, body mass index; LD-PSC, large-duct-PSC; IBD, inflammatory bowel disease; AST, aspartate aminotransferase; ULN, upper limit of normal; γGT, gamma-glutamyl transpeptidase; ALP, alkaline phosphatase; PT, prothrombin time. Levels of significance are indicated in the table (Mann-Whitney test for continuous variables; Chi-squared test, or Fisher test where appropriate, for nominal variables). A *p* value of less than 0.05 was considered significant.

Table S6. Serum bile acids in patients with conserved gallbladders *vs.* cholecystectomy

	**Conserved gallbladder** (n=50)		**Cholecystectomy** (n=11)		**p**
**Total bile acids**, μmol/L	35.41	(12.75-149.50)	104.00	(13.88-247.40)	0.40
**Primary BA**, μmol/L	5.22	(2.15-58.12)	40.09	(3.87-123.40)	0.20
**CA**, μmol/L	2.39	(0.68-24.86)	12.96	(1.01-39.87)	0.25
**CDCA**, μmol/L	2.61	(1.34-28.68)	14.73	(2.62-41.30)	0.16
**CA/CDCA**	0.68	(0.38-1.17)	0.48	(0.35.1.13)	0.91
**Secondary BA**, μmol/L	0.55	(0.09-1.50)	0.43	(0.00-1.53)	0.78
**DCA**, μmol/L	0.31	(0.09-1.35)	0.41	(0.00-1.42)	0.88
**LCA**, μmol/L	0.07	(0.00-0.28)	0.04	(0.00-0.19)	0.72
**UDCA**, μmol/L	24.10	(7.86-84.67)	63.87	(8.71-123.70)	0.32
**Primary/secondary BA**	12.39	(2.82-39.49)	47.53	(3.21-412.45)	0.39
**Glycoconjugates**, μmol/L	24.80	(10.29-131.16)	83.53	(12.52-146.80)	0.41
**Tauroconjugates**, μmol/L	1.96	(0.41-20.93)	18.73	(1.09-45.63)	0.28
**Tauro-/glycoconjugates**	0.08	(0.03-0.22)	0.18	(0.07-0.26)	0.30
**UDCA/total BA**	0.70	(0.62-0.85)	0.62	(0.53-0.69)	0.06
**Glycoconjugates/total BA**	0.78	(0.69-0.87)	0.80	(0.65-0.80)	0.84
**Tauroconjugates/total BA**	0.07	(0.02-0.17)	0.14	(0.07-0.20)	0.29
**Hydrophobicity index**	0.77	(0.74-0.86)	0.82	(0.75-0.87)	0.37
**FGF-19,** pg/mL	129.77	(72.53-217.76)	322.44	(322.44-322.44)	0.26
**C4,** ng/mL	1.20	(0.20-2.00)	0.30	(0.30-0.30)	0.70

Quantitative variables are expressed as medians (interquartile range). Abbreviations: BA, bile acid; CA, cholic acid; CDCA, chenodeoxycholic acid; DCA, Deoxycholic acid; LCA, lithocholic acid; UDCA, Ursodeoxycholic acid. Levels of significance are indicated in the table (Mann-Whitney test). A *p* value of less than 0.05 was considered significant.
