# Supplementary material for: Protective potential of the gallbladder in primary sclerosing cholangitis
Source: JHEP Rep. 2022 Dec 17;5(4):100649. doi: 10.1016/j.jhepr.2022.100649 (PMC10009728; doi:10.1016/j.jhepr.2022.100649)
Supplement: Multimedia component 2 [file mmc2.docx]

**JHEP Reports**

**CTAT methods**

Tables for a “Complete, Transparent, Accurate and Timely account” (CTAT) are now mandatory for all revised submissions. The aim is to enhance the reproducibility of methods.

- Only include the parts relevant to your study
- Refer to the CTAT in the main text as ‘Supplementary CTAT Table’
- Do not add subheadings
- Add as many rows as needed to include all information
- Only include one item per row

**If the CTAT form is not relevant to your study, please outline the reasons why:**

|  |
| --- |

- 1. **Antibodies**

| **Name** | **Citation** | **Supplier** | **Cat no.** | **Clone no.** |
| --- | --- | --- | --- | --- |
| anti-CK19 | Jacob F  Proceedings of the National Academy of Sciences of the United States of America 77.7 (1980 Jul): 4113-7. | Developmental Studies Hybridoma Bank, Iowa University, IA, USA |  | TROMA III |
| anti-F4/80 | Ko M  Dis Model Mech 14:N/A (2021) | Abcam, Cambridge, UK |  | SP115 |
|  |  |  |  |  |
|  |  |  |  |  |
|  |  |  |  |  |

- 1. **Cell lines**

| **Name** | **Citation** | **Supplier** | **Cat no.** | **Passage no.** | **Authentication test method** |
| --- | --- | --- | --- | --- | --- |
| **NA** |  |  |  |  |  |

- 1. **Organisms**

| **Name** | **Citation** | **Supplier** | **Strain** | **Sex** | **Age** | **Overall n number** |
| --- | --- | --- | --- | --- | --- | --- |
| **NA** |  |  |  |  |  |  |

- 1. **Sequence based reagents**

Please see Table S1

| **Name** | **Sequence** | **Supplier** |
| --- | --- | --- |
|  |  |  |

- 1. **Biological samples**

| **Description** | **Source** | **Identifier** |
| --- | --- | --- |
| NA |  |  |

- 1. **Deposited data**

| **Name of repository** | **Identifier** | **Link** |
| --- | --- | --- |
| **NA** |  |  |

- 1. **Software**

| **Software name** | **Manufacturer** | **Version** |
| --- | --- | --- |
| Statistical Package for the Social Science | IBM SPSS Statistics, Chicago, IL, USA | version 24 |
| GraphPad | GraphPad Software, San Diego, CA, USA | version 5 |

- 1. **Other (*e.g*. drugs, proteins, vectors etc.)**

| **NA** |  |  |
| --- | --- | --- |
|  |  |  |

- 1. **Please provide the details of the corresponding methods author for the manuscript:**

| **Sara Lemoinne,**  [sara.lemoinne@aphp.fr](mailto:sara.lemoinne@aphp.fr)  Assistance Publique – Hôpitaux de Paris, Sorbonne University, Department of Hepatology, Saint-Antoine Hospital,  184 rue Faubourg Saint Antoine, 75012 Paris, France  Phone: +33 1 49282923 Fax: +33 149282107 |
| --- |

**2.0 Please confirm for randomised controlled trials all versions of the clinical protocol are included in the submission. These will be published online as supplementary information.**

| **NA** |
| --- |
